# Machine Learning Models for 3-Month Outcome Prediction Using Radiomics of Intracerebral Hemorrhage and Perihematomal Edema from Admission Head Computed Tomography (CT)

**DOI:** 10.3390/diagnostics14242827

**Published:** 2024-12-16

**Authors:** Fiona Dierksen, Jakob K. Sommer, Anh T. Tran, Huang Lin, Stefan P. Haider, Ilko L. Maier, Sanjay Aneja, Pina C. Sanelli, Ajay Malhotra, Adnan I. Qureshi, Jan Claassen, Soojin Park, Santosh B. Murthy, Guido J. Falcone, Kevin N. Sheth, Seyedmehdi Payabvash

**Affiliations:** 1 Department of Radiology and Biomedical Imaging, Yale School of Medicine, New Haven, CT 06510, USAjakob.sommer@rwth-aachen.de (J.K.S.); anh.trn@yale.edu (A.T.T.);; 2 Department of Neurology, University Medicine Göttingen, 37075 Göttingen, Germany; ilko.maier@med.uni-goettingen.de; 3 Department of Otorhinolaryngology, University Hospital of Ludwig Maximilians Universität München, 81377 Munich, Germany; 4 Department of Radiation Oncology, Yale School of Medicine, New Haven, CT 06510, USA; 5 Feinstein Institute for Medical Research, Manhasset, New York, NY 11030, USA; 6 Department of Neurology, Zeenat Qureshi Stroke Institute, University of Missouri, Columbia, MO 65211, USA; 7 Department of Neurology, New York-Presbyterian Hospital, Columbia University Irving Medical Center, Columbia University, New York, NY 10065, USA; 8 Department of Biomedical Informatics, Columbia University Vagelos College of Physicians & Surgeons, New York, NY 10032, USA; 9 Department of Neurology, Weill Cornell School of Medicine, New York, NY 10065, USA; 10 Department of Neurology, Yale School of Medicine, New Haven, CT 06510, USA; 11 Center for Brain and Mind Health, Yale School of Medicine, New Haven, CT 06510, USA; 12 Department of Radiology, New York-Presbyterian Hospital, Columbia University Irving Medical Center, Columbia University, New York, NY 10065, USA

**Keywords:** intracerebral hemorrhage, perihematomal edema, radiomics, machine learning

## Abstract

**Background**: Intracerebral hemorrhages (ICH) and perihematomal edema (PHE) are respective imaging markers of primary and secondary brain injury in hemorrhagic stroke. In this study, we explored the potential added value of PHE radiomic features for prognostication in ICH patients. **Methods**: Using a multicentric trial cohort of acute supratentorial ICH (*n* = 852) patients, we extracted radiomic features from ICH and PHE lesions on admission non-contrast head CTs. We trained and tested combinations of different machine learning classifiers and feature selection methods for prediction of poor outcome—defined by 4-to-6 modified Rankin Scale scores at 3-month follow-up—using five different input strategies: (a) ICH radiomics, (b) ICH and PHE radiomics, (c) admission clinical predictors of poor outcomes, (d) ICH radiomics and clinical variables, and (e) ICH and PHE radiomics with clinical variables. Models were trained on 500 patients, tested, and compared in 352 using the receiver operating characteristics Area Under the Curve (AUC), Integrated Discrimination Index (IDI), and Net Reclassification Index (NRI). **Results**: Comparing the best performing models in the independent test cohort, both IDI and NRI demonstrated better individual-level risk assessment by addition of PHE radiomics as input to ICH radiomics (both *p* < 0.001), but with insignificant improvement in outcome prediction (AUC of 0.74 versus 0.71, *p* = 0.157). The addition of ICH and PHE radiomics to clinical variables also improved IDI and NRI risk-classification (both p < 0.001), but with a insignificant increase in AUC of 0.85 versus 0.83 (*p* = 0.118), respectively. All machine learning models had greater or equal accuracy in outcome prediction compared to the widely used ICH score. **Conclusions**: The addition of PHE radiomics to hemorrhage lesion radiomics, as well as radiomics to clinical risk factors, can improve individual-level risk assessment, albeit with an insignificant increase in prognostic accuracy. Machine learning models offer quantitative and immediate risk stratification—on par with or more accurate than the ICH score—which can potentially guide patients’ selection for interventions such as hematoma evacuation.

## 1. Introduction

Acute intracerebral hemorrhages (ICH) represent one of the most devastating types of strokes, with up to 80% rate of mortality and morbidity [1]. The mechanical injury from hematoma and subsequent expansion serve as the primary mechanism of injury to the brain tissue, followed by a cascade of neuroinflammatory reaction to neurotoxic byproducts from hemoglobin degradation that leads to secondary brain injury and manifests by perihematomal edema (PHE) [2,3,4]. Previous studies have identified PHE volume as a potential independent contributor to unfavorable outcomes following ICH [1]. From a neuroimaging standpoint, hematoma and surrounding edema are respective representatives of primary and secondary brain injuries and, thus, serve as main imaging prognostic variables of acute ICH.

Radiomics refers to the extraction of quantitative features representing the shape, texture, and intensity of target lesions from medical images. These features have been shown to provide valuable biological correlates regarding underlying tissue pathology. The admission non-contrast and angiographic CT radiomics have also proven as a reliable prognostic marker in ischemic and hemorrhagic stroke [5,6]. Previous studies have demonstrated a stronger association between the admission CT radiomics of hematoma with clinical outcome than ICH volume [5]. Although the association of ICH and PHE radiomics with clinical outcomes has been demonstrated separately [7,8], a knowledge gap remains regarding models that leverage the combined prognostic value of ICH and PHE radiomics for outcome prediction.

In this study, we aimed to determine the added value of combining the radiomic features from ICH and PHE, as respective imaging markers of primary and secondary brain injury, to predict 3-month clinical outcome. We used a large multicentric trial dataset of patients with hypertensive supratentorial ICH to develop and validate prognostic models using radiomics features extracted from hematoma and surrounding edema to predict 3-month outcome. We trained, optimized, and validated different combinations of machine learning and feature selection models for prediction of functional outcome with inputs from ICH radiomics alone, ICH and PHE radiomics, baseline clinical variables, and a combination of ICH and PHE radiomics with clinical variables. We also compared the prognostic performance of machine learning models with ICH score [9], the most widely validated clinical tool for risk-stratification in ICH patients [10].

## 2. Materials and Methods

### 2.1. Ascertainment of Study Subjects

**Figure 1:**
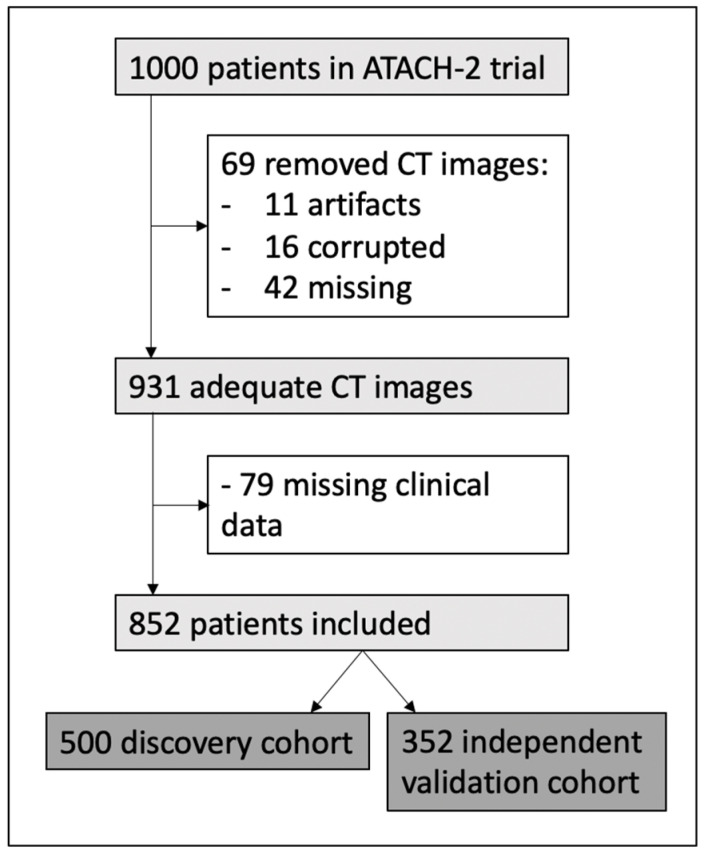
Flowchart of patients that were included in our study.

We used the Antihypertensive Treatment of Acute Cerebral Hemorrhage II (ATACH-2) Trial dataset for this study [11]. The ATACH-2 was a multicenter, randomized, open-label trial comparing the efficacy of intensive blood pressure reduction versus standard treatment in patients with acute supratentorial ICH, at least one systolic blood pressure of ≥180 mmHg, and hematoma volume < 60 mL [11]. For our analysis, we excluded subjects with a poor quality of head CTs and missing clinical information (Figure 1). The study protocol for the trial and current analysis was approved in corresponding centers’ institutional review boards.

### 2.2. Segmentation of ICH and PHE on Non-Contrast CTs

Both hematoma and surrounding edema were manually segmented on axial slices of admission non-contrast head CT scans, using MRIcron (associated with NeuroDebian, BSD Licence, University of South Carolina, Columbia, SC, USA) and 3D-Slicer (Brigham and Women’s Hospital, Boston, MA, USA; BSD-style open source license). Separate segmentation masks were generated for ICH and PHE by trained research associates [12], and then reviewed and refined by an expert neuroradiologist (SP). We applied a 1-to-40 Hounsfield unit (HU) threshold for PHE, and up to 200 HU threshold for ICH segmentations, as described previously [12,13,14]. Distinction of PHE from pre-existing leukoaraiosis and small vessel disease remains technically challenging. We delineated the neuroanatomical distribution of hypodensity around the hematoma to differentiate PHE from leukoaraiosis, which typically has a symmetric pattern in the periventricular and deep white matter regions. In addition, a subset of scans was segmented again to determine the inter- and intra-rater reliability of radiomics features and restrict the analysis to those features with consistency across inter- and intra-rater segmentations.

### 2.3. Extraction of Radiomic Features

We adapted and applied a customized pipeline for pre-processing of head CTs and radiomic feature extraction pipeline based on pyradiomics version 2.2.0 [15,16]. To compensate for slice thickness and matrix size differences across different scans, we resample all scans to isotropic 1 mm voxel dimensions. Subsequently, we extracted *n* = 14 “shape”, *n* = 18 “first-order”, and *n* = 75 “texture-matrix” features from original CT scans followed by eight “coif-1” wavelet decompositions and three Laplacian of Gaussian (LoG) filter image derivatives (at 2 mm, 4 mm, and 6 mm sigma) for the first-order and texture features—resulting in a total of 1130 ICH and 1130 PHE radiomics features [15] (Supplementals Table S1).

### 2.4. Models’ Input and Outcome

We trained and tested models with five different input strategies: (1) “ICH” using 1130 radiomic of hematoma lesions alone; (2) “ICH + PHE” using 2260 radiomic features from ICH and PHE lesions, separately; (3) “clinical” using clinical predictors of outcome at the time of admission (Supplemental Table S2) [17]; (4) “ICH + clinical” using 1130 radiomic of hematoma lesion and clinical variables and; (5) “ICH/PHE + clinical” using 2260 radiomic features from ICH and PHE as well as clinical variables to predict outcomes. The modified Rankin Scale (mRS) at three-month follow-up or closest interval available was used for clinical outcome. The models were trained to predict binarized outcome as favorable (0-to-3 mRS) versus poor (4-to-6 mRS), as commonly applied for ICH patients [11].

### 2.5. Training, Cross-Validation, and Independent Validation of Models

The data were split into training/cross-validation versus independent test cohort (Figure 1) by stratification. Feature selection, cross-validation, and optimization were exclusively performed in a training set, strictly isolating the test cohort from the training process. The process has been described previously [5,6]. Briefly, to ensure the robustness of radiomic features, we first calculated features’ intraclass correlation coefficients (ICC) in subset of scans with multiple segmentation and limited analysis to features with the lower bounds of 95% confidence intervals (CI) above 0.8, using the “psych” package in R. Then, we combined 6 different machine learning classifiers with 6 feature selection methods. Feature selection methods included: Minimum Redundancy Maximum Relevance (MRMR), Pearson Correlation-based Redundancy Reduction and Mutual Information Maximization Filter (pMIM), Ridge regression (RIDGE); Hierarchical Clustering (HClust), Principal Component Analysis-based feature selection (PCA), and no Feature Selection (noFS). Machine learning classifiers were Elastic Net-regularized logistic regression (ElNet), Random Forest (RF), Support Vector Machine with sigmoid kernel (SVM sig), Support Vector Machine with radial kernel (SVM rad), Naïve Bayes (NBayes), and XGBoost (XGB). For each of the 36 combinations, we optimized the number of features, and machine learning model parameters using Bayesian hyperparameter optimization (with the “rBayesianOptimization” package) through a repeated 5-fold cross-validation. Details of hyperparameters for each model are provided in Supplemental Table S3. Subsequently, we applied each model combination with optimized hyperparameters, performed 20× repeats of 5-fold for cross-validation performance, and determined the performance across validation folds using area under curve (AUC) of receiver operating characteristics (ROC). The average AUCs from 100 validation folds are reported for each model combination performance. Final model combinations of the machine learning classifier and feature selection method were trained on whole training/cross-validation cohorts using optimized hyperparameters and then tested in the independent test cohort. Notably, given the small number of clinical variables, we applied no feature selection in models using clinical variables alone as input.

### 2.6. Model Failure Analysis

We compared the clinical and imaging variables between patients with favorable outcome (true positive and false positive) versus poor outcome (true negative and false negative) of correct and wrong predictions by the model in the independent test cohort using Student’s t-test for continuous variables, the Mann–Whitney rank sum test for ordinal variables, and Fisher’s exact test for binary categorical variables. This analysis (Table 1) delineates potential differences between subjects in whom the model combination performed accurately versus those with prediction failure.

### 2.7. Risk Assessment Plots

To examine the risk assessment capabilities of a new model to an existing one, some investigators have introduced Integrated Discrimination Index (IDI) and Net Reclassification Index (NRI) [17,18]. These indices quantify the ability to correctly reclassify events and non-events for both models. The NRI estimates the proportion of patients reclassified to a more appropriate risk category, and IDI quantifies the slope improvement of the discriminant curves. These indices can provide additional information to AUC analysis. We used the “predictABEL” R package to calculate NRI and IDI for comparison of different models and generate the risk assessment plots for visual depiction of NRI and IDI differences.

### 2.8. Comparison with “ICH Score”

The “ICH score” is the most widely used and validated clinical grading scale to assess the severity and predict the prognosis of ICH patients. The ICH score ranges from 0 to 6 and is the sum of individual points assigned based on admission GCS score (2 points for ≤ 4 and 1 point for 5-to-12); age ≥ 80 years (1 point); infratentorial origin (1 point); hematoma volume ≥ 30 mL (1 point), and presence of intraventricular hemorrhage (IVH, 1 point). We calculated the ICH score for all subjects in independent test cohort and compared its predictive performance with each of the five machine learning models.

### 2.9. Statistical Analysis

We presented continuous variables as mean ± standard deviation, summarized ordinal variables as median with interquartile range, and reported nominal variables as frequency with accompanying percentage. To compare training/cross-validation versus test cohorts, we utilized Student’s t-test for continuous variables, the Mann–Whitney rank sum test for ordinal variables, and Fisher’s exact test for binary categorical variables. All statistical analyses were executed using R version 3.6.3. For statistical comparisons of paired AUCs, we applied DeLong’s test and calculated associated p-values and AUC (95% CI), using the “pROC” package in R.

## 3. Results

### 3.1. Patients’ Characteristics

The flowchart in Figure 1 depicts the inclusion process for 852 patients in our analysis. We then randomly split patients into training/cross-validation (*n* = 500) versus independent test (*n* = 352) cohorts. In Supplemental Table S4 we compared the clinical characteristics between these two study cohorts, which showed no significant difference. The supplemental Table S5 summarizes the clinical characteristics between those with poor versus favorable outcome in the study population (*n* = 852). Favorable outcome (mRS 0-to-3) was associated with younger age, less severe symptoms at admission, smaller hematoma and edema volumes, and absence of intraventricular hemorrhage. The mean intra- and inter-rater ICC for ICH segmentation volumes were 0.94, and 0.92, and for PHE were 0.94 and 0.93, respectively.

### 3.2. Best Performing Models for Outcome Prediction

**Figure 2:**
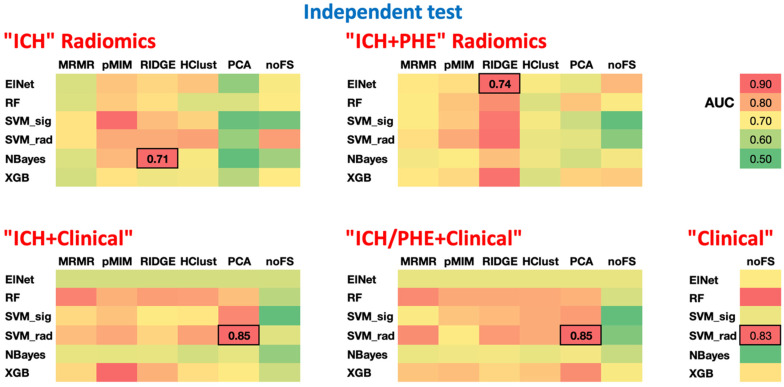
Heatmap AUCs of different combinations of machine learning classifiers (listed in rows) and feature selection methods (in column). The best preforming model with the highest AUC is marked with bold font and borders. A green color refers to an AUC of 0.5, yellow to 0.7, and red to 0.9 as summarized in the cohort bar in upper right corner. AUC = area under curve of receiver operating characteristic analysis; ElNet = Elastic Net-regularized logistic regression; HClust = Hierarchical Clustering; MRMR = Minimum Redundancy Maximum Relevance; NBayes = Naïve Bayes; noFS = no Feature Selection; PCA = Principal Component Analysis-based feature selection; pMIM = Pearson Correlation-based Redundancy Reduction with Mutual Information Maximization Filter; RF = Random Forest; RIDGE = Ridge regression; SVM rad = Support Vector Machine with radial kernel; SVM sig = Support Vector Machine with sigmoid kernel; XGB = XGBoost.

**Figure 3:**
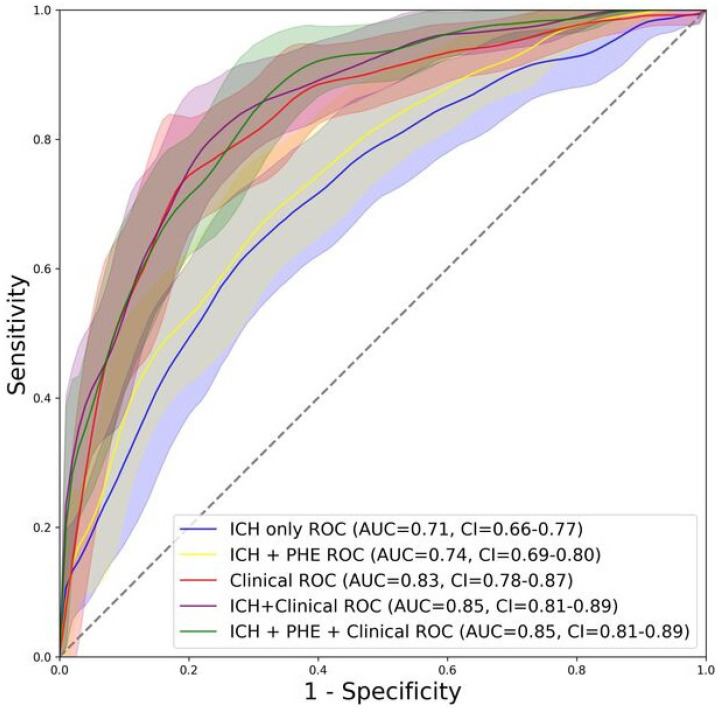
The ROC curves of the best performing ICH radiomics (NBayes—RIDGE), ICH + PHE radiomics (ElNet—RIDGE), radiomics + clinical (SVM rad—PCA), Clinical only (noFS—SVM rad) and ICH + Clinical (SVM rad—PCA) models in predicting poor outcomes at 3-month follow-up and their integrated Sensitivities.

Figure 2 depicts the heatmap of different model combinations’ performance for prediction of poor outcome in independent test cohort. Detailed average AUCs from the cross-validation process and the AUCs of the final optimized models in independent test cohorts are included in supplemental Figure S1. Using ICH radiomics alone, the NBayes and RIDGE combination achieved the best outcome prediction with AUC = 0.71 (95% CI: 0.65–0.77) in the independent test cohort (Supplemental Table S6). Using ICH + PHE radiomics, the Elastic Net and RIDGE combination achieved the best performance with AUC = 0.74 (95% CI: 0.70–0.79) in the independent test cohort, without significant difference from ICH radiomics alone (*p* = 0.157) (Supplemental Table S7). Using clinical variables alone, the SVM rad model achieved the best prediction with AUC = 0.83 (95% CI: 0.78–0.87) in the independent test cohort, which was higher than ICH + PHE radiomics (*p* = 0.004) and ICH radiomics (*p* < 0.001). The best performing ICH + Clinical model with inputs from ICH radiomics and clinical variables was the combination of SVM rad and PCA, achieving an AUC = 0.85 (95% CI 0.81–0.89), which was not significantly different from the Clinical model (*p* = 0.07) but higher than the ICH + PHE and ICH radiomics models (*p* < 0.001). Finally, adding baseline clinical variables to ICH + PHE radiomics as a model input (ICH/PHE + clinical), the SVM rad and PCA combination achieved the best performance with AUC = 0.85 (95% CI 0.81–0.89) in the independent test cohort, which was significantly higher than the ICH + PHE radiomics and ICH radiomics models (both *p* < 0.001) but not significantly different from the clinical (*p* = 0.118) or ICH + Clinical model (*p* = 0.750). Figure 3 shows ROC curves and their integrated Sensitivities of the best performing models in comparison.

### 3.3. Comparison Risk Assessment Plots of the Best Performing Models’

**Figure 4:**
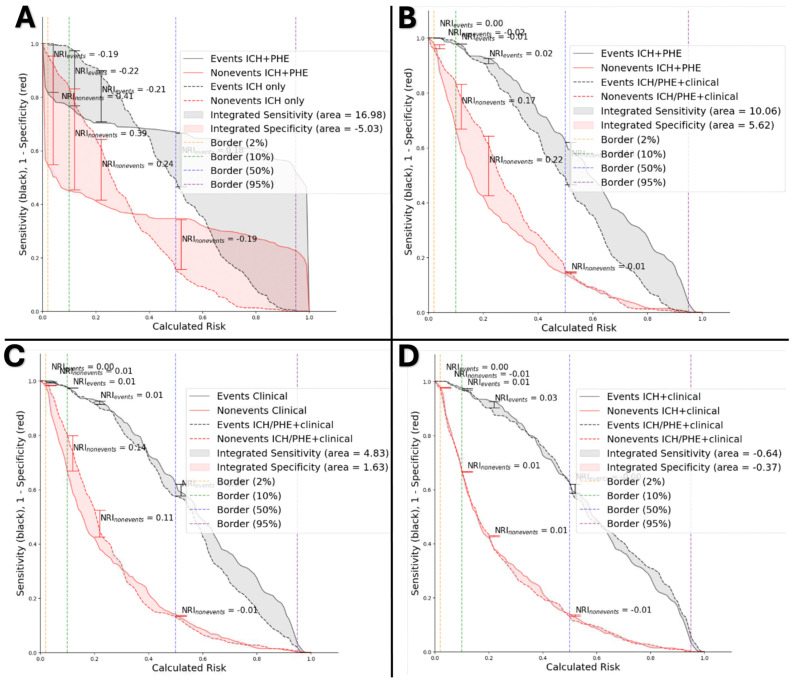
Comparing the best performing models. (**A**) ICH radiomics versus ICH + PHE radiomics, (**B**) ICH + PHE radiomics versus ICH/PHE + clinical models, (**C**) clinical versus ICH/PHE + clinical models, and (**D**) ICH + clinical versus ICH/PHE + clinical.

In risk assessment analysis, the ICH + PHE radiomics input model compared to ICH radiomics model (Figure 4A), demonstrated a 17% improvement in NRI (*p* < 0.001), and 11.95% increase in IDI (*p* < 0.001). The ICH/PHE + clinical risk assessment compared to ICH + PHE radiomics (Figure 4B) input showed 37.7% improvement in NRI and 3.73% increase in IDI (*p* < 0.001). The ICH/PHE + clinical compared to the clinical model (Figure 4C) demonstrated a 28% improvement in NRI, and the 6.5% increase in IDI (*p* < 0.001). The ICH/PHE + clinical compared to ICH + clinical model (Figure 4D) demonstrated a 3.9% improvement in NRI and a 1.01% increase in IDI (*p* < 0.001).

### 3.4. Failure Analysis of Models’ Prediction

The failure analysis of the select models is summarized in Table 1. Wrong predictions were more likely in patients with favorable outcome than those with poor outcomes in ICH and ICH + PHE radiomic models (*p* < 0.001); however, in ICH/PHE + clinical and ICH + clinical models, wrong predictions were more likely in those with poor outcome (*p* = 0.015, *p* = 0.036, respectively). The analysis across all five models indicates that overall patients with favorable outcomes who were incorrectly predicted tend to exhibit significantly larger hematoma and edema volumes and more severe symptoms (higher NIHSS and lower GCS) compared to those correctly predicted. In contrast, among those with poor outcomes, the patients who were incorrectly predicted exhibited significantly smaller hematoma and edema volumes and less severe symptoms (lower NIHSS and higher GCS) compared to those with correct predictions.

### 3.5. Comparing Machine Learning Models with the ICH Score

The ROC analysis of the ICH score demonstrates an AUC of 0.688 for predicting poor outcomes (mRS score of 4 to 6), which was significantly lower than the clinical, ICH + clinical, and ICH/PHE + clinical models (all *p* values < 0.001). However, it was not significantly different from the ICH radiomics (*p* = 0.424) and ICH + PHE radiomics (*p* = 0.081) models.

## 4. Discussion

In this study, we applied a rigorous and methodological framework of cross-validation and independent testing to explore the added prognostic value of PHE radiomics to ICH features from admission non-contrast head CT for prediction of 3-month clinical outcome. We also examined radiomics-based models with those including a combination of clinical and radiomic features. In both cross-validation and independent testing, although the addition of PHE radiomic features to those of ICH had statistically insignificant increase in prognostic accuracy, it still improved the IDI and NRI metrics for individual-level risk assessment. Similarly, when comparing multimodal models that incorporate both clinical and radiomics inputs with those of clinical variables alone, we found improvements in IDI and NRI metrics, but without a significant difference in AUC. The IDI and NRI results suggest that the inclusion of radiomic features alongside clinical variables—or the addition of PHE radiomics to ICH radiomics (with or without clinical variables)—has improved predicted probabilities of poor versus favorable outcomes at the individual level, even though the overall ranking of predictions, as measured by AUC, did not change significantly. Since AUC reflects the overall ranking of predictions, improvements in individual-level risk estimates—captured by IDI and NRI—will not significantly affect AUC unless the ranking of patients changes sufficiently to improve outcome classification. Nonetheless, improvements in risk estimates may be particularly valuable when focusing on high-risk subcohorts of ICH patients.

We also demonstrated that machine learning models using clinical variables (with or without radiomic features) are more accurate in predicting outcomes than the widely used ICH score. Additionally, radiomics-based models achieved prognostic accuracy on par with the ICH score. The primary practical benefit of such radiomics-based models is their potential for fast, quantitative risk stratification. With automated ICH and PHE segmentation, these radiomics-based risk stratification tools can quickly identify patients likely to have a poor prognosis immediately after the baseline head CT detects ICH. Subsequently, additional clinical information, physical examinations, and laboratory tests can improve prognostication once they become available. Then, the addition of radiomics to clinical variables in machine learning models can improve individual-level risk estimation, with the practical benefit becoming most relevant in high-risk sub cohorts. Of note, the final model, with optimized hyperparameters, runs on the independent test cohort (*n* = 352) in just 26 s, highlighting the machine learning tools’ potential for efficient treatment guidance in urgent clinical settings.

It is noteworthy that the list of features selected for inclusion in the final best performing ICH + PHE radiomics model show that shape metrics of hematoma as well as both ICH and PHE texture features are included; however, PHE volumetric feature were not selected. Specifically, hematoma maximum coronal diameter and the smallest axis length of an enclosing ellipsoid were predictors of outcome. This suggests that shape metrics of ICH as well as texture of both ICH and PHE on admission non-contrast head CT provide more prognostically relevant information than baseline PHE volumetric variables.

Prediction failure in models with radiomics input was more likely among patients with favorable outcomes, whereas the addition of clinical variables made wrong prediction less likely among those with favorable outcome. Thus, a combination of models can provide more balanced risk assessment in ICH patients.

Our study extends beyond prior research focused solely on individual PHE metrics, as highlighted by Levine et al.’s [19] observation of an association between higher absolute PHE volume and decreased 90-day mortality, along with similar findings from other studies [20,21] employing relative PHE volume indicators to predict functional outcomes post-ICH. It should, however, be noted that the prognostic relevance of PHE volumetric measures may become stronger in the subacute phase as secondary brain injury progresses, as is suggested by some prior studies [22,23].

Recently, multiple studies applied admission CT radiomics to predict hematoma expansion and ICH outcomes [6,24,25,26]; however, to the best of our knowledge, this is the first study to include both ICH and PHE radiomics for outcome prediction. Given that ICH and PHE, respectively, represent areas of primary and secondary brain injury, we hypothesized that combination of radiomics features from these two lesions would provide more robust prognostication [27].

Huang et al. have recently reported an AUC of 0.714 for prediction of 3-month poor outcome using PHE radiomics features on admission CT [7]. Combining PHE radiomics with hematoma volume and clinical variables, they achieved an AUC of 0.91 in outcome prediction [7]. However, in their dataset, the average hematoma and edema volumes among patients with poor outcomes were three times larger than those with favorable outcomes [7]. This three-fold difference in hematoma volumes between outcome cohorts was much greater than in our dataset, where the average ICH and PHE volumes in patients with poor outcomes were less than twice those observed in patients with favorable outcomes. This inherently more drastic difference between those with poor versus favorable outcomes in their study has likely contributed to higher AUC of their multimodal model combining PHE radiomics with hematoma volume and clinical variables [7].

Our study has multiple strengths. We utilized a large, homogenous, and prospectively collected dataset of supratentorial ICH patients. The outcome measures were methodologically gathered as part of the clinical trial design. We applied a rigorous feature selection and machine learning training process with hyperparameter optimization followed by independent testing on a cohort that was strictly isolated from the training/optimization process to prevent any data leakage. The ICH and PHE radiomics features included in the final model provide insight into the inner workings of the model decision making and the relative importance of these features in the prediction process. Finally, model bias analysis revealed that our models tend to overestimate the likelihood of poor outcome.

The two main etiologies of spontaneous ICH are chronic hypertension and cerebral amyloid angiopathy [28]. Our study is inherently limited by the inclusion criteria of ATACH-2 trial, which only enrolled patients with supratentorial ICH who had at least one systolic blood pressure reading above 180 mmHg [11]. As a result, the main mechanism of ICH in the majority of our patients was likely hypertensive, as evidenced by the high proportion of deep hemorrhages compared to lobar ones. Among consecutive ICH patients, nearly half present with deep hemorrhages, which are the typical imaging manifestation of hypertensive cerebral small vessel disease. Although the ATACH-2 trial did not establish a definitive etiology for ICH, it is important to note that our study cohort was more likely to have hypertensive ICH, which may limit the generalizability of our findings to patients with cerebral amyloid angiopathy and lobar ICH. In addition, differentiating hypodensities on head CT that are due to PHE from other etiologies, such as prior lacunar infarcts [29,30], cerebral small vessel disease, leukoaraiosis, or previous neuroinflammatory lesions, presents a technical limitation. In our dataset, brain MRIs were not available to provide a clear distinction of PHE. Therefore, for PHE segmentation, we relied on the distribution of hypodensity, which surrounds the hyperdense ICH and appears slightly denser than pre-existing chronic white matter injury. While non-contrast CT scans are crucial in initial stroke imaging due to its accessibility, CT perfusion metrics provides a superior tool for predicting hemorrhagic transformation by evaluation perfusion parameters [31]. Notably, the patients in our dataset had smaller hematoma volumes and better outcomes—with lower rates of morbidity and mortality—compared to general population of ICH patients [11], which limit the generalizability of our model. This is reflected in the higher baseline GCS scores observed in our cohort, as the ATACH-2 trial predominantly included patients with less severe presentations.

## 5. Conclusions

We found that the addition of radiomic features from PHE to those from hemorrhagic lesions, as well as the inclusion of radiomics with clinical risk-factors, can improve the individual-level risk assessment of machine learning models, although the overall increase in outcome prediction accuracy was statistically insignificant. We also demonstrated that machine learning models, including those using input from hemorrhagic lesion radiomics alone, can achieve prediction accuracy equal to or higher than the widely used ICH score. The practical benefit of such models is providing immediate risk stratification after the completion of the admission head CT, which can potentially guide patient selection for neurosurgical interventions such as hematoma evacuation.

## Figures and Tables

**Table 1:** Failure analysis of the different models in prediction of 3-month poor outcome.

ICH Radiomics	Favorable Outcome			Poor Outcome		
	Correct Prediction (*n* = 120)	Wrong Prediction (*n* = 98)	*p*-Value	Correct Prediction (*n* = 104)	Wrong Prediction (*n* = 30)	*p*-Value
ICH Volume (mL)	4.3 ± 3.8	13.5 ± 9.6	<0.001	18.2 ± 12.9	4.7 ± 4.6	<0.001
PHE Volume (mL)	0.8 ± 0.96	1.9 ± 1.9	<0.001	2 ± 2.6	0.8 ± 0.8	0.014
CT slice Thickness (mm)	5 ± 1.5	5 ± 1.9	1	5 ± 2	5 ± 1.2	1
Sex (male)	77 (64%)	71 (72%)	0.243	59 (57%)	15 (50%)	0.538
NIHSS	6 (1–11)	11 (4–17)	<0.001	17 (8–26)	10 (6.25–13.75)	<0.001
GCS	15 (15–15)	15 (13–17)	1	14 (10–18)	15 (14–16)	0.032
Age (years)	61.5 ± 10.6	58 ± 12.9	0.029	64.5 ± 14.5	57 ± 14.6	0.014
**ICH and PHE Radiomics**	**Favorable Outcome**			**Poor Outcome**		
	**Correct Prediction** **(*n* = 121)**	**Wrong Prediction** **(*n* = 97)**	***p*-Value**	**Correct Prediction** **(*n* = 107)**	**Wrong Prediction** **(*n* = 27)**	***p*-Value**
data	data	data				
ICH Volume (mL)	4.6 ± 5.1	12.3 ± 10.2	<0.001	17.3 ± 13.3	10.4 ± 10.2	0.013
PHE Volume (mL)	0.8 ± 1.2	1.8 ± 1.9	<0.001	1.9 ± 2.6	1.1 ± 1.4	0.126
CT slice Thickness (mm)	5 ± 1.5	4.9 ± 1.9	0.665	5 ± 1.9	5 ± 1.7	1
Sex (male)	78 (64%)	70 (72%)	0.246	57 (53%)	17 (63%)	0.395
NIHSS	6 (0–12)	10 (3–17)	<0.001	17 (9.5–24.5)	9 (3–15)	<0.001
GCS	15 (15–15)	15 (13–17)	1	14 (10–18)	14 (12–16)	1
Age (years)	61 ± 11.3	59 ± 12.7	0.221	65 ± 13.8	57 ± 14.6	0.009
**Clinical Only**	**Favorable Outcome**			**Poor Outcome**		
	**Correct Prediction** **(*n* = 179)**	**Wrong Prediction** **(*n* = 39)**	***p*-Value**	**Correct Prediction** **(*n* = 100)**	**Wrong Prediction** **(*n* = 34)**	***p*-Value**
ICH Volume (mL)	7.2 ± 8.7	12.7 ± 9.6	0.007	16.2 ± 13.9	13.8 ± 10.7	0.360
PHE Volume (mL)	1.0 ± 1.6	1.7 ± 1.7	0.015	1.8 ± 2.6	1.5 ± 1.9	0.533
CT slice Thickness (mm)	5 ± 1.7	4.9 ± 1.8	0.742	5 ± 1.9	5 ± 1.2	1
Sex (male)	124 (69%)	24 (62%)	0.351	51 (51%)	23 (68%)	0.112
NIHSS	7 (2–12)	15 (6.5–23.5)	<0.001	17 (8–26)	9 (2.25–15.75)	<0.001
GCS	15 (15–15)	14 (9.5–18.5)	<0.001	14 (10–18)	15 (14–16)	0.027
Age (years)	59 ± 11.9	65 ± 10.8	0.004	68.5 ± 13.7	57 ± 13.2	<0.001
**ICH Radiomics ** **+ Clinical**	**Favorable Outcome**			**Poor Outcome**		
	**Correct Prediction** **(*n* = 172)**	**Wrong Prediction** **(*n* = 46)**	***p*-Value**	**Correct Prediction** **(*n* = 106)**	**Wrong Prediction** **(*n* = 28)**	***p*-Value**
ICH Volume (mL)	6.9 ± 7.2	13.4 ± 11.7	<0.001	16.7 ± 13.9	13.5 ± 9.9	0.255
PHE Volume (mL)	1.1 ± 1.5	1.6 ± 1.9	0.056	1.8 ± 2.5	1.2 ± 2	0.243
CT slice Thickness (mm)	5 ± 1.5	5 ± 1.99	1	5 ± 1.8	5 ± 1.6	1
Sex (male)	119 (69%)	29 (63%)	0.478	58 (55%)	16 (57%)	0.835
NIHSS	7 (2–12)	14 (4.25–23.75)	<0.001	17 (8.25–25.75)	9 (3.75–14.25)	<0.001
GCS	15(14.75–15–25)	14 (11.25–16.75)	<0.001	14 (10–18)	15 (14–16)	0.042
Age (years)	59 ± 11.7	64 ± 13	0.013	66 ± 13.7	60 ± 15.2	0.046
**ICH and PHE Radiomics ** **+ Clinical**	**Favorable Outcome**			**Poor Outcome**		
	**Correct Prediction** **(*n* = 181)**	**Wrong Prediction** **(*n* = 37)**	***p*-Value**	**Correct Prediction** **(*n* = 93)**	**Wrong Prediction** **(*n* = 41)**	***p*-Value**
ICH Volume (mL)	7.1 ± 7.9	13.3 ± 11.6	<0.001	17.1± 14.2	12.3 ± 9.85	0.185
PHE Volume (mL)	1.1 ± 1.5	1.7 ± 2.1	0.041	1.9 ± 2.7	1.2 ± 1.5	0.122
CT slice Thickness (mm)	5 ± 1.5	4.9 ± 1.8	0.722	5 ± 1.9	5 ± 1.7	1
Sex (male)	126 (69.6%)	22 (60%)	0.249	49 (53%)	25 (61%)	0.452
NIHSS	8 (3–13)	14 (4–24)	0.537	18 (9–27)	10 (4–16)	0.261
GCS	15 (14–16)	14 (11–17)	0.722	14 (10–18)	15 (13–17)	0.742
Age (years)	59 ± 12	64 ± 11.1	0.020	67 ± 13.5	63 ± 14.8	0.128

Favorable outcome defined by a 3-month modified Rankin Score ≤ 3. GCS = Glasgow Coma Scale; NIHSS = NIH Stroke Scale; PHE = Perihematomal Edema.

## Data Availability

The raw data supporting the conclusions of this article will be made available by the authors on request.

## Supplementary Materials

The following supporting information can be downloaded at: https://www.mdpi.com/article/10.3390/diagnostics14242827/s1, Supplemental Table S1: Parameters for radiomic features extraction; Supplemental Table S2: Clinical variables; Supplemental Table S3: Machine learning hyperparameters; Supplemental Table S4: Patients Characteristics for training/cross-validation vs independent test Cohort; Supplemental Table S5: Comparison of patients with favorable versus poor outcomes; Supplemental Table S6: List of ICH radiomics in final “ICH Radiomics” model; Supplemental Table S7: List of ICH and PHE radiomics in final “ICH + PHE” model; Supplemental Figure S1: Different models’ performances.
