# Supplementary material for: Machine Learning Models for 3-Month Outcome Prediction Using Radiomics of Intracerebral Hemorrhage and Perihematomal Edema from Admission Head Computed Tomography (CT)
Source: Diagnostics (Basel). 2024 Dec 16;14(24):2827. doi: 10.3390/diagnostics14242827 (PMC11674633; doi:10.3390/diagnostics14242827)
Supplement: Supplementary file 1 [file diagnostics-14-02827-s001.zip › diagnostics-3325595-supplementary.pdf]

Supplemental Materials:

Supplemental Table S1: Parameters for radiomic features extraction. **2**

Supplemental Table S2: Clinical variables. **3**

Supplemental Table S3: Machine learning hyperparameters. **4**

Supplemental Table S4: Patients Characteristics for training/cross-validation vs independent test Cohort **5**

Supplemental Table S5: Comparison of patients with favorable versus poor outcomes. **6**

Supplemental Table S6: List of ICH radiomics in final "ICH Radiomics" model **7**

Supplemental Table S7: List of ICH and PHE radiomics in final "ICH+PHE" model **8**

Supplemental Figure S1: Different models' performances. **9**

Supplemental Table S1: Parameters for radiomic features extraction.

**Parameter File**
Image type:
Original: {}
LoG: sigma: [2.0, 4.0, 6.0]
Wavelet: {}
Settings:
Interpolator: 'sitkLinear’
Resampled Pixel Spacing: [1, 1, 1]
Resegment Range: [1.0, 40.0]
Pad Distance: 10
Bin Width: 2
Label: 1

Pyradiomics.yaml configuration and parameter file for the extraction of the ICH and PHE parameter file. The Hounsfield Unit (HU) Range was defined as 0 to 40 HU for PHE and 0 to 100 for ICH.

Supplemental Table S2: Clinical variables.

**Clinical Variables**	
Demographic Data:	- Age<br>- Sex<br>- Race (Hispanic, black, white)<br>- GCS<br>- NIHSS
Physical exam:	- Blood pressure (systolic and diastolic)<br>- Treatment
Laboratory test results:	- Platelet<br>- PTT (Partial thromboplastin time)<br>- INR (Prothrombin Time)<br>- Glucose<br>- White blood cell count<br>- Hemoglobin<br>- Hematocrit<br>- Natrium<br>- Kalium<br>- Blood urea nitrogen<br>- Creatinine
Clinical history:	- Stroke CNS<br>- Cardiovascular history<br>- Diabetes mellitus
Localization:	- Basal ganglia<br>- Thalamus<br>- Lobar<br>- Side hemorrhage
Other:	- Hydrocephalus<br>- Pineal gland shift<br>- PHE volume<br>- Hematoma volume<br>- Septum shift<br>- Intraventricular hemorrhage

The list of physical exam, demographic data, clinical history and laboratory test results that were included as clinical variable in models with “clinical”, “ICH+clinical”, and “ICH/PHE+clinical” inputs.

Supplemental Table S3: Machine learning hyperparameters.

Machine learning	Number of rounds	Hyper-parameter	Lower bound	Upper bound	Type
**ElNet**	100	nFeatures	2	30	integer
		alpha	0	1	numeric
**RF**	150	nFeatures	2	30	integer
		mtry	2	40	integer
		maxnodes	2	32768	integer
**XGB**	200	nFeatures	2	30	integer
		eta	0	1	numeric
		gamma	0	10	numeric
		max_depth	3	15	integer
		min_child_weight	0	20	numeric
		subsample	0.4	1	numeric
		colsample_bytree	0.4	1	numeric
		lambda	0.5	1	numeric
**SVM_sig**	200	nFeatures	2	30	integer
		gamma	0	0.5	numeric
		coef0	0	1	numeric
		cost	0.1	10	numeric
**NBayes**	50	nFeatures	2	30	integer
**SVM_rad**	150	nFeatures	2	30	integer
		gamma	0	0.5	numeric
		cost	0.1	10	numeric

The list and range of hyperparameter for different machine learning classifiers that were finetuned during Bayesian Optimization in cross-validation framework

Supplemental Table S4: Patients Characteristics for training/cross-validation vs independent test Cohort

	Training Cohort (n=500)	Test Cohort (n=352)	P value
*Favorable outcome **	318 (64%)	218 (62%)	0.6657
Age (years)	62.4 ± 13.1	62.2 ± 13.2	0.8269
Sex (male)	299 (60%)	222 (63%)	0.3538
Race White	133 (27%)	101 (28.7%)	0.5331
Race Asian	267 (53%)	182 (52%)	0.7273
Race Black	65 (13%)	39 (11%)	0.4571
Hispanic ethnicity	35 (7%)	30 (8%)	0.4332
Admission NIHSS	12 (5 – 19)	12 (5 – 19)	1
Admission GCS	14 (12 – 15)	14 (12 – 15)	1
Intensive Treatment	257 (51%)	172 (49%)	0.4869
Systolic blood pressure (mmHg)	174.5 ± 24.1	174.9 ± 25.6	0.8162
Hematoma volume (mL)	13.8 ± 12.8	13.3 ± 11.6	0.5598
Intraventricular hemorrhage	366 (73%)	254 (72%)	0.7549
Basal ganglia hemorrhage	281 (56%)	200 (57%)	0.8885
Thalamus hemorrhage	155 (31%)	116 (33%)	0.551
Lobar hemorrhage	63 (13%)	36 (10%)	0.3288
PHE Volume (mL)	2.2 ± 3.1	1.9 ± 2.0	0.1107

Favorable outcome defined by a 3-month modified Rankin Score ≤3

GCS= Glasgow Coma Scale; IQR=interquartile; NIHSS= NIH Stroke Scale; PHE= perihematomal edema

Supplemental Table S5: Comparison of patients with favorable versus poor outcomes.

	**Favorable Outcome (mRS 0-to-3)**	**Poor Outcome (mRS 4-to-6)**	**P value**
Age (years)	59.9 ± 12.3	66.4 ± 13.5	< 0.001
Male sex	348 (65%)	173 (55%)	0.004
Race White	131 (24%)	103 (33%)	0.012
Race Asian	306 (57%)	143 (45%)	< 0.001
Race Black	59 (11%)	45(14%)	0.177
Race Hispanic	40 (7%)	25 (8%)	0.815
Admission NIHSS (median, IQR)	9 (6)	16 (7)	< 0.001
Admission GCS (median, IQR)	14 (2)	13 (3)	< 0.001
Intensive Treatment	265 (49%)	158 (50%)	0.875
SBP prior randomization (mmHg)	175 ± 23	174 ± 28	0.738
Hematoma volume (ml)	10.6 ± 9.36	18.7 ± 14.7	< 0.001
Intraventricular hemorrhage	81 (15%)	135 (42%)	< 0.001
Basal ganglia	306 (57%)	175 (55%)	0.628
Thalamus	159 (30%)	112 (35%)	0.084
Lobar hemorrhage	70 (13%)	29 (9%)	0.076
Edema Volume (ml)	1.8 ± 2.3	2.6 ± 3.1	< 0.001

Comparison of clinical, demographic, and imaging characteristics of patients with favorable (3-month modified Rankin scale, mRS, of 0-to-3) versus those with poor outcomes (3-month mRS of 4-to-6) in whole dataset.

Supplemental Table S6: List of ICH radiomics in final "ICH Radiomics" model
List of hematoma lesion radiomic features selected and included in the best performing model combination (NBayes – RIDGE) for outcome prediction. The feature importance scores

**Feature selected for the poor outcome prediction model**	**Feature importance score**
wavelet.HLL_glcm_Imc2	-0.03851
log.sigma.4.0.mm.3D_gldm_LargeDependenceEmphasis	0.297645805
wavelet.LLH_glrlm_RunLengthNonUniformity	0.212966167
wavelet.LLH_glrlm_RunVariance	0.163524804
log.sigma.4.0.mm.3D_glrlm_RunLengthNonUniformityNormalized	0.12928789
wavelet.HLL_glcm_Imc1	0.120745076
wavelet.HHL_glcm_Imc1	0.069507094
log.sigma.6.0.mm.3D_firstorder_Kurtosis	0.064083769
log.sigma.4.0.mm.3D_glcm_Idmn	0.06259871
wavelet.LHL_glcm_Imc2	0.057198307
wavelet.HLL_glcm_Correlation	0.036141246
wavelet.LLH_glcm_Imc1	0.00610027
wavelet.LHH_glrlm_RunEntropy	0.005677193
original_shape_Maximum2DDiameterColumn	0.003881088
log.sigma.6.0.mm.3D_glcm_Correlation	0.003591706
wavelet.LLL_ngtdm_Busyness	0.000703293
log.sigma.2.0.mm.3D_firstorder_10Percentile	-0.001093645
wavelet.LLH_glcm_MCC	-0.00743612
original_firstorder_90Percentile	-0.01833604
original_glcm_Imc1	-0.032300491
original_shape_LeastAxisLength	-0.035475024
wavelet.LLH_glszm_ZoneEntropy	-0.04428918
log.sigma.2.0.mm.3D_glcm_Idmn	-0.047484763
original_ngtdm_Busyness	-0.067805293
wavelet.HHL_glcm_Imc2	-0.076588223
log.sigma.4.0.mm.3D_glrlm_RunPercentage	-0.0798848
log.sigma.4.0.mm.3D_gldm_DependenceVariance	-0.10557376
log.sigma.4.0.mm.3D_glcm_Idn	-0.115588275
wavelet.LLH_glrlm_LongRunEmphasis	-0.22695916
log.sigma.4.0.mm.3D_glrlm_ShortRunEmphasis	-0.24946318

represent the absolute values of our most important features from the highest to lowest value

Supplemental Table S7: List of ICH and PHE radiomics in final "ICH+PHE" model

ICH and PHE radiomic features selected for prediction of poor outcome	Feature importance score
log.sigma.6.0.mm.3D_firstorder_Skewness (PHE)	-0.34845816
original_shape_Maximum2DDiameterColumn (ICH)	0.245758586
wavelet.HLL_glcm_Imc2 (ICH)	-0.238513914
log.sigma.2.0.mm.3D_glcm_InverseVariance (PHE)	0.200506461
wavelet.HLL_glcm_Correlation (ICH)	-0.163061871
log.sigma.4.0.mm.3D_glrlm_RunLengthNonUniformityNormalized (ICH)	-0.156746166
original_firstorder_90Percentile (ICH)	-0.149717018
log.sigma.4.0.mm.3D_glrlm_ShortRunEmphasis (ICH)	-0.137110122
log.sigma.2.0.mm.3D_glcm_MaximumProbability (PHE)	-0.121425748
log.sigma.4.0.mm.3D_glrlm_RunPercentage (ICH)	-0.113768365
log.sigma.4.0.mm.3D_glcm_MaximumProbability (PHE)	-0.106223113
log.sigma.4.0.mm.3D_glszm_SmallAreaLowGrayLevelEmphasis (PHE)	-0.10405325
original_shape_LeastAxisLength (ICH)	0.097571085
log.sigma.6.0.mm.3D_glrlm_ShortRunLowGrayLevelEmphasis (PHE)	-0.082169031
wavelet.LLL_ngtdm_Busyness (ICH)	0.078854773
log.sigma.6.0.mm.3D_glcm_Correlation (ICH)	0.076240942
log.sigma.4.0.mm.3D_gldm_LargeDependenceLowGrayLevelEmphasis (PHE)	-0.075471983
log.sigma.4.0.mm.3D_gldm_LowGrayLevelEmphasis(edema)	-0.070802728
log.sigma.4.0.mm.3D_gldm_LargeDependenceEmphasis (ICH)	0.06591448
log.sigma.4.0.mm.3D_glrlm_LowGrayLevelRunEmphasis (PHE)	-0.062964542
log.sigma.4.0.mm.3D_glszm_LowGrayLevelZoneEmphasis (PHE)	-0.060477208
wavelet.LHL_glcm_Imc2 (ICH)	-0.05858678
log.sigma.4.0.mm.3D_glrlm_LongRunLowGrayLevelEmphasis (PHE)	-0.050912957
log.sigma.6.0.mm.3D_gldm_LowGrayLevelEmphasis (PHE)	-0.050761042
log.sigma.2.0.mm.3D_glszm_GrayLevelNonUniformityNormalized (PHE)	-0.045134261
original_ngtdm_Busyness (ICH)	0.041137567
log.sigma.4.0.mm.3D_glcm_JointEnergy (PHE)	-0.041108221
log.sigma.6.0.mm.3D_glrlm_LowGrayLevelRunEmphasis (PHE)	-0.040875549
log.sigma.4.0.mm.3D_glcm_Idmn (ICH)	0.022986799
log.sigma.2.0.mm.3D_firstorder_10Percentile (ICH)	0.010205789

List of hematoma and edema lesion radiomic features selected and included in the best performing model combination (ElNet – RIDGE) for outcome prediction ("ICH+PHE" model). The feature importance scores represent the absolute values of our most important features from the highest to lowest value.

Cross-validation

"ICH" Radiomics

	MRMR	pMIM	RIDGE	HClust	PCA	noFS
ElNet	0.72	0.73	0.73	0.70	0.65	0.73
RF	0.72	0.73	0.72	0.69	0.68	0.73
SVM_sig	0.72	0.71	0.73	0.70	0.65	0.56
SVM_rad	0.71	0.72	0.73	0.69	0.65	0.71
NBayes	0.69	0.70	0.70	0.67	0.59	0.68
XGB	0.72	0.72	0.72	0.71	0.69	0.74

"ICH+PHE" Radiomics

	MRMR	pMIM	RIDGE	HClust	PCA	noFS
ElNet	0.73	0.76	0.76	0.69	0.68	0.76
RF	0.73	0.74	0.74	0.72	0.68	0.76
SVM_sig	0.73	0.71	0.76	0.68	0.63	0.55
SVM_rad	0.73	0.75	0.75	0.69	0.63	0.51
NBayes	0.70	0.70	0.72	0.64	0.61	0.67
XGB	0.73	0.74	0.75	0.73	0.68	0.76

AUC
0.90
0.80
0.70
0.60
0.50

"ICH+Clinical"

	MRMR	pMIM	RIDGE	HClust	PCA	noFS
ElNet	0.75	0.75	0.75	0.75	0.75	0.75
RF	0.84	0.84	0.84	0.84	0.84	0.78
SVM_sig	0.84	0.84	0.84	0.84	0.83	0.58
SVM_rad	0.84	0.84	0.84	0.84	0.83	0.74
NBayes	0.80	0.80	0.80	0.77	0.78	0.69
XGB	0.84	0.84	0.84	0.85	0.85	0.79

"ICH/PHE+Clinical"

	MRMR	pMIM	RIDGE	HClust	PCA	noFS
ElNet	0.75	0.75	0.75	0.75	0.75	0.75
RF	0.84	0.84	0.84	0.84	0.84	0.78
SVM_sig	0.84	0.84	0.83	0.83	0.82	0.54
SVM_rad	0.84	0.83	0.82	0.82	0.82	0.50
NBayes	0.80	0.80	0.79	0.78	0.78	0.68
XGB	0.84	0.84	0.84	0.85	0.83	0.84

"Clinical"

	noFS
ElNet	0.82
RF	0.79
SVM_sig	0.81
SVM_rad	0.81
NBayes	0.73
XGB	0.80

Independent test

"ICH" Radiomics

	MRMR	pMIM	RIDGE	HClust	PCA	noFS
ElNet	0.66	0.69	0.68	0.69	0.63	0.67
RF	0.66	0.69	0.68	0.66	0.66	0.68
SVM_sig	0.68	0.71	0.69	0.68	0.61	0.61
SVM_rad	0.68	0.70	0.70	0.70	0.63	0.70
NBayes	0.66	0.69	**0.71**	0.67	0.60	0.63
XGB	0.66	0.68	0.67	0.67	0.64	0.68

"ICH+PHE" Radiomics

	MRMR	pMIM	RIDGE	HClust	PCA	noFS
ElNet	0.70	0.70	**0.74**	0.69	0.66	0.72
RF	0.70	0.71	0.73	0.64	0.71	0.69
SVM_sig	0.69	0.71	0.74	0.68	0.61	0.42
SVM_rad	0.70	0.72	0.74	0.66	0.65	0.49
NBayes	0.68	0.69	0.71	0.67	0.62	0.68
XGB	0.70	0.70	0.74	0.63	0.71	0.71

AUC
0.90
0.80
0.70
0.60
0.50

"ICH+Clinical"

	MRMR	pMIM	RIDGE	HClust	PCA	noFS
ElNet	0.77	0.77	0.77	0.77	0.77	0.77
RF	0.85	0.83	0.84	0.84	0.83	0.74
SVM_sig	0.82	0.83	0.82	0.82	0.84	0.63
SVM_rad	0.83	0.84	0.83	0.84	**0.85**	0.78
NBayes	0.79	0.79	0.79	0.77	0.79	0.69
XGB	0.82	0.83	0.83	0.82	0.82	0.76

"ICH/PHE+Clinical"

	MRMR	pMIM	RIDGE	HClust	PCA	noFS
ElNet	0.77	0.77	0.77	0.77	0.77	0.77
RF	0.84	0.84	0.84	0.84	0.84	0.72
SVM_sig	0.82	0.83	0.84	0.84	0.84	0.42
SVM_rad	0.84	0.82	0.84	0.84	**0.85**	0.50
NBayes	0.78	0.79	0.80	0.77	0.80	0.68
XGB	0.83	0.83	0.83	0.83	0.84	0.81

"Clinical"

	noFS
ElNet	0.82
RF	0.83
SVM_sig	0.81
SVM_rad	0.83
NBayes	0.74
XGB	0.82

Supplemental Figure S1: Different models' performances.

The average ROC AUCs from validation folds in repeated 5-fold cross-validation process and the AUC's of the final optimized models in the independent test cohort.
